# Sustainability of hospital-based midwife-led antenatal care consultation — a qualitative study

**DOI:** 10.1186/s12913-021-06863-w

**Published:** 2021-08-23

**Authors:** Anja Siegle, Friederike Zu Sayn-Wittgenstein, Martina Roes

**Affiliations:** 1 Department of Nursing Science, University of Witten/Herdecke, Alfred-Herrhausen-Straße 50, 58448 Witten, Germany; 2 Faculty of Business Management and Social Sciences, University of Applied Sciences Osnabrueck, Network of Midwifery, P.O. Box 19 40, 49009 Osnabrück, Germany

**Keywords:** Midwife-led antenatal consultation, Implementation, Qualitative study, Sustainability

## Abstract

**Background:**

All evidence-based knowledge and improvement strategies for quality care must be implemented so patients can benefit from them. In Germany, national expert standards (NES) contribute to quality care in nursing and midwifery. The NES defines for several action levels a dedicated level of quality, which is operationalized by structure, process, and outcome (SPO) criteria. An NES to promote normal childbirth was developed and implemented in 2014. The first action level involves midwife-led antenatal consultation (ML-AC) being conducted in a structured way. Most implementation processes aim to accomplish sustainability, but implementation studies rarely use definitions or a theory of sustainability, even when assessing sustainability. This lack led to the assumption that intervention sustainability after implementation is still a largely unexplored domain. The aim of this study is to investigate the sustainability of midwife-led antenatal consultation (ML-AC) implemented at two hospitals, in Germany.

**Methods:**

In a qualitative approach, 34 qualitative interviews were conducted (between March and October 2017) using semi-structured interview guides. All interviews were transcribed verbatim, anonymized and analyzed thematically using framework method. Four groups of stakeholders in two hospitals offering ML-AC were interviewed: managers (*n* = 8), midwives conducting ML-AC (*n* = 10), pregnant women who attended ML-AC (*n* = 8), and physicians working in obstetrics (*n* = 8) at the hospitals.

**Results:**

The interviewees identified key benefits of ML-AC on a personal and unit level, e.g., reduced obstetric interventions, easier admission processes. Furthermore, the participants defined key requirements that are needed for routinized and institutionalized ML-AC, such as allocating working time for ML-AC, and structural organization of ML-AC. All study participants saw a need to establish secure long-term funding. Additionally, the interviewed staff members stated that ML-AC topics need evaluating and adapting in the future.

**Conclusions:**

Implementing ML-AC in the hospital setting has led to manifold benefits. However, reimbursement through the health care system will be needed to sustain ML-AC.

Hospitals implementing ML-AC will need to be aware that adaptations of the NES are necessary to accomplish routinization and institutionalization. After initial implementation, sustaining ML-AC will generally require on-going monitoring and evaluating of benefits, routinization/institutionalization and further development.

## Introduction

### Background

Research in medicine, but also in nursing and midwifery science, is permanently generating new and evidence-based knowledge. This knowledge should be used to contribute to improve therapy and care for patients. But generating this knowledge alone is not enough to sustainably increase the quality of care for patients because all evidence-based knowledge, new procedures and improvement strategies must first be transferred from theory to practice [1]. Thus, there is a need for implementation. Implementation is defined as a constellation of processes that are intended to put an intervention into use within an organization [2, 3].

Implementation processes need to be evaluated to determine whether they were sufficiently successful or not. There are many possible outcome measures that can be used to determine degree of success, e.g., adoption, acceptability, appropriateness, fidelity, maintenance, sustainability [4–7]. Grol and Grimshaw showed that, if improvements of an intervention are not sustained, then the result will be a waste of scarce resources, as well as stakeholders’ time. There will be no lasting gain for patients [8]. Therefore, sustainability of an intervention is a key implementation outcome [7] and a priority topic for implementation science [9–11]. Furthermore, there is a need for “more consistent and complete reporting of sustainability to ensure that lessons learned can be of direct benefit to future implementation of interventions” [12].

Even though sustainability has been examined in the health care context, it has rarely been defined, even in studies assessing sustainability [10, 13]. Frequently, sustainability has been measured only after a short timeframe of several months to a year. The later-stage challenges of sustaining interventions have received little attention [9, 10]. In particular, intervention sustainability is still a largely unexplored domain in nursing [6] and midwifery [14].

### Promotion of normal childbirth

According to the maternal health community all over the world, the use of medical interventions in childbirth is increasing and, if inappropriately used, is a sign of poor-quality care [15–20]. Excessive or routine use of obstetric interventions leads to over-medicalization during childbirth [15]. Therefore, to promote normal childbirth, different guidelines [21, 22], and a framework for quality maternal and newborn care [23] have been developed worldwide. The framework for quality maternal and newborn care aims to describe the contribution of midwifery to the quality of care of women and infants globally. It defines effective practices to promote normal processes for childbearing women and infants, e.g., upright positions, immersion in water, relaxation techniques for pain relieve during birthing, which can be introduced in midwife-led antenatal consultation (ML-AC) [23]. In Germany, 98% of all children are born in hospitals in which midwives and physicians share the responsibility for the woman giving birth [24]. Although midwives can provide prenatal care most pregnant women attend an obstetric physician for prenatal care, resulting in women arriving in labor at the hospital without having the opportunity to meet a midwife before. Midwives offer childbirth and postpartum education courses on antenatal and labor care for pregnant women. Additionally, midwives provide postpartum care at home for mother and child up to twelve weeks, which is reimbursed by the health insurance.

National nursing expert standards (NES) have been developed to define quality care in Germany. An NES is structured along the steps of the nursing process. For each action level structure, process, and outcome (S-P-O) criteria on quality care are defined [25]. In 2014, the first NES by midwives to **improve the physiological childbirth process,** which includes ML-AC, was introduced [26]. The main goal of the NES on physiological childbirth is that every pregnant woman receives tailored individual support (see Table 1) from a midwife to promote normal childbirth [26]. Physiological birth means that normal childbirth is defined as a birth with no or as little as possible well founded medical and pharmacological interventions [26]. Structured and systematic counseling of a pregnant woman during pregnancy by a midwife allows normal birth to be promoted and prepared for, but was not part of hospital-based routine antenatal care in Germany. The NES to promote physiological childbirth recommends a hospital-based ML-AC [26]. Key requirements of this structured and systematic ML-AC [26] are summarized in Table 1.

**Table 1:** Example elements describing ML-AC according to the NES to promote physiological birth

•	The midwife invites the pregnant woman to reflect about her wishes, expectations, and anxieties concerning the upcoming birth and to ask questions.
•	The midwife assesses individual needs of the pregnant woman, encourages trust in the woman’s ability to give birth, explains the birth phases emphasizing the latency phase, and if necessary, imparts knowledge on birth giving.
•	An individual birth plan can be the result of the consultation.

The conceptual basis of each NES also includes guidance on how an NES can be implemented [27]. In the NES method paper, model implementation as part of developing an NES follows a systematic process: the 4-step implementation model [27]. The 4-step implementation model contains a description of prerequisites and the following steps for implementation: 1. training, 2. adaptation, 3. introduction and 4. audit. In Step One, an educational meeting and training classes on the NES are offered. Step Two contains tailoring and adaptation of the NES content to the specific hospital. Step Three comprises a guided introduction and application of the NES content in practice. Step Four includes an evaluation of the NES in this. In the model, implementation of feasibility and facilitators and barriers are analyzed, and if necessary the content is adapted before final publication of the NES [27].

Even though the 4-step implementation model provides detailed information on implementing an NES, hospital managers have to structure and conduct the implementation themselves. Often, the implementation succeeds only to a certain degree [5, 28]. It is largely unknown to what degree implementation of ML-AC according to NES succeeded and whether implementation sustainability has been achieved in the hospitals. Successful implementation is, however, not simply determined by results obtained directly after implementation; it requires the standard to be sustained. Without sustainability, an NES has failed. Hence, it is of importance to explore intervention sustainability of ML-AC as defined in the NES.

### Sustainability as an important implementation outcome measure

A definition and theoretical foundation are a prerequisite to identifying key dimensions that influence sustainability and highlight how those key dimensions differ and under what circumstances. In their concept analysis, Fleiszer et al. (2015) offer a broad conceptualization of sustainability that extends beyond program institutionalization and/or program benefits alone. Sustainability is defined as “a process that emerges from and succeeds innovation implementation, wherein improvements are maintained, new ways of working become routine, surrounding systems are transformed in support, and the innovation may even be developed, over a period of time appropriate to a given situation” [6]. The authors describe three characteristics of sustainability of health care innovations: (1) benefits, (2) routinization and institutionalization, and (3) development. The first term benefit means the consistent achievement of goals, including lasting improvement in positive patient outcomes, and continued perception of accomplishment [6, 29, 30]. The second characteristic of sustainability is institutionalization and routinization of an intervention. In their concept analysis, the authors found heterogeneous and synonymous use of these terms. According to Fleiszer et al. (2015), institutionalization implies the concretization of organizational infrastructure and integration of change in subsystems, such as established committees or dedicated budgets [6]. Routinization involves cycles of repeated action in social structure, e.g., practice and organizational routines [6]. This characteristic involves the embedding of structures and processes into the habitual practices of individuals, organizations, and systems [6, 29, 31]. The third characteristic relates to development. Fleiszer et al. (2015) describe development as the continual enhancement of users’ abilities and resources to maintain an innovation [6, 29, 32, 33].

### Measuring sustainability after implementation of NES ML-AC

All three characteristics (benefits, institutionalization/routinization, development) are relevant to determining sustainability of ML-AC [6]. Fleiszer’s sustainability model is chosen since it considers a broad characterization of sustainability and it helps to recognize the variety of influencing factors at multiple levels as well as characteristics and factors that may determine the level of innovation sustainability over time [6]. Describing perceived benefits may help midwives intending to implement ML-AC to enable evidence-based practice.

Described real-world experience of conditions or factors, which are context-, leadership- and process-related in hospitals, can help to plan and prepare for sustainable implementation.

### Aim and research questions

Examining intervention sustainability of ML-AC according to NES is part of a bigger study. The overall aim of the larger study is to explore an ML-CA’s implementation process and the outcomes fidelity, penetration and sustainability. A two-phase explanatory sequential mixed-methods design was selected since it allows complex concepts to be studied using first a quantitative and then a qualitative strand to explain results and explore them further [34, 35]. The quantitative strand comprised a quantitative document analysis of ML-AC documents and is published elsewhere [36]. The qualitative strand contained semi-structured interviews with pregnant women, midwives, managers and physicians. The advantage of this design is that the qualitative phase can be refined and designed on what is learned from the initial phase [35].

The aim of this paper is to present a part of the larger study including results on the sustainability of ML-AC according to NES. Three research questions were defined for this part: (1.) What kind of benefits as reported by participants can be seen in ML-AC? (2.) Which structures and processes have been embedded in the hospital or have helped to ensure routinization and institutionalization? (3.) What development is needed to sustain ML-AC in the future? This paper presents the results of an empirically grounded study of the sustainability characteristics of ML-AC in two hospitals. This study contributes to challenges that occur during an implementation period at a later-stage and how these impact sustaining interventions. Furthermore, the study provides detailed information on the degree of implementation success of ML-AC in midwifery.

## Methods

### Design

A qualitative design was chosen based on the three research questions specified to explore sustainability in detail from the perspective of different stakeholders connected with ML-AC according to NES. As methodological orientation, pragmatism was chosen because it allows one to study what works in everyday life. Pragmatism repudiates the metaphysical concept of truth, but rather covers the existence of single or multiple realities that are available to empirical study [35].

### Setting

Two hospitals who have implemented the ML-AC into their obstetrics unit were approached by the 1st author and agreed to participate. Hospital A has implemented the ML-AC to promote physiological childbirth in 2014. The hospital has a physician-led delivery unit and 2400 births a year. In hospital B, there is a physician-led, as well as a midwife-led, delivery unit with altogether 1700 births a year. Since 2011, midwives in hospital B offer the ML-AC for pregnant women wishing to give birth in the midwife-led delivery unit. There are only 18 midwife-led delivery units opposed to 187 physician-led delivery units all over Germany [24]. In hospital B women can choose to give birth in the midwife-led delivery unit given there are no pathologies or birthing risks identified. The goal of the ML-AC for pregnant women who prefer to deliver in the midwife-led delivery unit is to offer prenatal care and to ensure that women meet eligibility criteria for midwife-led care. Additionally, since 2015, the midwives of hospital B offer the ML-AC to promote normal childbirth to pregnant women delivering in the physician-led delivery unit.

### Recruitment of participants

In a convenience sample all managers, midwives, and physicians in both hospitals were invited to participate. In total 34 participants were interviewed: managers, midwives conducting the ML-AC, pregnant women who benefitted from the ML-AC, and physicians working in obstetrics at the hospitals. To ensure anonymity, the head midwife, head of nursing department, head physician, and persons from the quality insurance department from each hospital were grouped as “managers”. Midwives asked pregnant women at the beginning of the ML-AC if they agreed to take part in the study. All study participants were included if written informed consent was given.

### Data collection

Based on the literature, semi-structured interview guides [37] -- one each for managers, midwives, pregnant women, and physicians -- were developed containing open-ended questions around benefits, routinization and institutionalization, developments, and the future of the ML-AC (for examples, see Table 2). Each interview guide was discussed in a group of qualitative researchers and pilot-tested.

**Table 2:** Example questions of interview guide for midwives conducting the ML-AC

Structure	Example question for midwives
Open query	You are offering ML-AC for pregnant women here in the hospital. Tell me what is it all about?
Specific query	What is your goal of ML-AC? What kind of benefits do you see? What is needed to offer ML-AC in the future?
Ending	Is there something important we did not talk about and you would like to add?

Between March and October 2017, the 1st author, with experience in qualitative interviews and nursing and who was unknown to participants conducted all face-to-face interviews in German. Reflective postscript notes were taken after each interview [38]. The interviews were conducted with women on the same day after having the ML-AC appointment as an outpatient service in their last trimester of pregnancy. Average interview duration was 54 min (range 23–100 min) with managers, 38 min (range 28–55 min) with midwives, 28 min (range 20–33 min) with physicians and 19 min (range 5–29 min) with pregnant women.

### Data analyses

All interviews were audio-recorded, transcribed verbatim [39], and anonymized. For data organization and to support analysis, transcripts were uploaded in MAXQDA 12 (Release 12.3.6). The data analysis was done according to Gale et al. (2013) using the following steps of framework method: 1) familiarization, 2) coding, in ways both inductive (open coding to identify new themes) and deductive (by theory-predefined codes), 3) development and application of an analytic framework, 4) data-charting in a matrix, and 5) interpretation [40]. Codes, themes, and the analytic framework were discussed with other qualitative researchers at different stages on a regular basis.

An ethics approval had been obtained from the ethics committee of the German Society for Nursing Science (Deutsche Gesellschaft für Pflegewissenschaft, No. 2018–1). The study was performed in accordance with the ethical standards as laid down in the 1964 Declaration of Helsinki and its later amendments. This paper follows the consolidated criteria for reporting qualitative research (COREQ) [41].

## Results

### Participant characteristics

Thirty-four interviews were conducted with managers (8), midwives (10), pregnant women (8), and physicians (8) in the two hospitals. The partners accompanied three interviewed pregnant women. All interviews took place within the hospital buildings, in line with participants’ preferences. Interviews with pregnant women were sometimes short due to time limitation of child care of other children. Study participant characteristics can be found in Table 3.

**Table 3:** Participant characteristics

	Managers	Midwives	Pregnant women	Physicians
**Number of participants**	8	10	8	8
**Gender**				
(men)	3	–	–	1
(women)	5	10	8	7
**Age**				
(average years)	45	42	32	39
(range years)	31–55	25–52	26–38	30–49
**Experience**				
(average years)	19	19	–	11
(range years)	6–27	3–30	–	4–19

### Results on sustainability

Results on sustainability characteristics 1.) perceived benefits, 2.) embedded structures and processes to ensure routinization and institutionalization, and 3.) development to sustain ML-AC in the future are described in the following. The authors have translated all quotes from German to English. All quotes are paraphrased.

### Characteristic of sustainability: (1) perceived benefits

Benefits include the consistent achievement of goals, lasting improvement in positive outcomes and continued perception of accomplishment [6, 29]. All participants reported substantial benefits from the ML-AC on a personal (woman, midwife, physician) or unit level (delivery unit). The perceived benefits, such as fewer medical interventions, increase of detailed information about women’s medical history and wishes, and easement of admission processes are presented in the following.

#### Benefits on a personal level

Midwives and physicians of both hospitals reported **fewer medical interventions** in childbirth. This is the major goal of the NES to promote physiological childbirth [26]. According to one physician in hospital A, they reduced their secondary caesarean section from 40 to 35% and sometimes even 30%. One midwife of hospital A pointed out: “We have fewer interventions, like forceps delivery, caesarean section, or vacuum extraction” (Hospital A-midwife 5). Managers, midwives, and physicians agreed that they have **more and better information** about the pregnant women. Midwives and physicians emphasized the general importance of gathering information before the woman arrives in labor pains. Applying the ML-AC allows information to be gathered about the medical history of the pregnant woman allows more time for in-depth questions and consultation. Also, the midwives and physicians pointed out that pregnant women are enabled to make decisions based on this information, e.g., having an episiotomy either performed or not. A midwife of hospital B emphasized: “Often a childbirth looks good in the health care documents, but the woman has anyway a trauma or something. This is a reason [for the pregnant woman] to come to ML-AC. Also acts of violence, death of a person, or relocation [author’s note: are reasons to take advantage of ML-AC].” (Hospital B-midwife 1).

Managers and midwives of both hospitals described how **worries and anxieties** of pregnant women can be addressed in the ML-AC. Addressing worries and anxieties of pregnant women also enables midwives of both hospitals to **empowering** pregnant women in their ability to give birth. In consequence, the pregnant women reported **a feeling of safety and trust** after the ML-AC, as intended by the midwives of both hospitals. One pregnant woman said that the midwives are very experienced, i.e., she trusted the midwife’s assessment during childbirth, and she assumed she endures more than she is expecting from herself. Managers and midwives of both hospitals described how the ML-AC documents enabled them to **match preferences** addressed by pregnant women with what may happen in the delivery unit. The birth plan, when not interfering with the safety of mother and child, now includes these preferences and serves as a guideline to support the midwives in both hospitals.

It seemed that the pregnant women felt more empowered and reported fewer worries and anxieties. Pregnant women in both hospitals appreciated the opportunity to ask questions, get detailed information, and have the paperwork done before childbirth. Furthermore, the pregnant women’s preferences could be better addressed, including her knowledge about changes that may occur under childbirth and which decisions to make. To explain their preferences and to ask questions in the ML-AC was an important source of relief for the pregnant women. One pregnant woman reported that her first baby was delivered by a secondary caesarean section. And now, after discussing her preferences during the ML-AC, she felt relieved and encouraged to try again to give vaginal birth: “This empowerment, simply empowerment. I had a caesarean section with my first child, an unplanned caesarean section, and now [after ML-AC] I feel encouraged to try anyway with my second child [to give vaginal birth].” (Hospital B-woman 4).

#### Benefit on a unit level

Managers and physicians of both hospitals and midwives of hospital B underlined an **easement in the admission process** when the pregnant woman came for childbirth. One manager of hospital A highlighted that time could be saved in both the admission process and in the time spent in the delivery unit. This manager stated that, in the ML-AC, women learn about the latency phase: in other words, women know better what to do and when to come to the hospital. According to this manager, this information saved time, since midwives and nurses had to monitor fewer women in first-stage labor. Another benefit concerning time saving, according to all participants, is that the documents and records for the childbirth are prepared and completed.

The identified benefits are not specific to each group of interviewed study participants. Statements about managers’ and midwives’ intention in offering the ML-AC and how women perceived several of the benefits are compared in Table 4, showing that pregnant women confirm the aims of the intended measures of the managers, midwives, and physicians.

**Table 4:** Benefits of ML-AC as reported by study participants

Benefit	Managers	Midwives	Physicians	Pregnant women
**Benefits on a personal level (midwife, physician, woman)**				
**Fewer medical interventions**		Fewer forceps deliveries, caesarean sections, or vacuum extractions	Reported reduction of caesarean sections	
**Quality of information**	Better information on pregnant woman	Better information - taken before labor pain start - better informed women	Better information on and better-informed pregnant women	Possibility to receive information and ask detailed questions without labor pain
**Worries, anxieties of pregnant women are addressed**	Worries and anxieties of the pregnant woman can be addressed	Worries and anxieties of the pregnant woman can be addressed		Worries and anxieties are addressed
**Empowerment of pregnant women**		To empower women to give birth naturally		Pregnant women feel empowered to give vaginal birth
**Increased feeling of safety for pregnant women**		To support a feeling of safety to the pregnant woman, being trustworthy		Feeling safe, having confidence in the midwife
**Expanded matching between preferences and services**	Service delivery matches the wishes of pregnant women while in the delivery unit	Service delivery matches the wishes of pregnant women while in the delivery unit		To gain influence on what happens in the delivery unit
**Benefit on a unit level (delivery unit)**				
**Easement in admission processes**	Time can be saved	Quick and easy admission, documents are prepared	Easy admission, documents are prepared	Relieved that medical history and birth plan are done

### Characteristic of sustainability: (2) institutionalization and routinization

Institutionalization and routinization involve the embedding of structures and processes of an intervention such as an NES into the habitual practices of individuals, organizations, and systems. Institutionalization implies the concretization of organizational infrastructure and integration of change in subsystems [6]. For successful institutionalization of the ML-AC, several structures and processes were crucial: staff budget, time slots for ML-AC appointments, and rooms. Additionally, structural organization and organizational infrastructure needed adaptation and embedding into the everyday practices of midwives and physicians (personal level), as well as on the unit (delivery unit) and organizational levels (hospital). These changes happened according to midwives, mangers and physicians over the last 2–3 years and were accomplished without additional time or resources being set aside for project management. Identified institutionalization and routinization will be described in the following.

#### Institutionalization

According to the managers of both hospitals, **staff budget** was increased and institutionalized to offer the ML-AC. In hospital A, the staff budget was initially organized in overtime of midwives. After this initial testing phase in overtime payment, staff budget was increased by hospital management according to the number of midwives needed. In hospital B, staff budget was at first increased from a donation and then by the hospital management. The midwives offering ML-AC in both hospitals emphasized the importance of having separate working schedules to offer the ML-AC. This ensures that, when there are too few midwives, they will not be called on for both, midwifery care in the delivery unit and ML-AC duty simultaneously.

In both hospitals, there was an increase in **time slots for the ML-AC appointments**. Starting with two mornings, hospital A extended time slots to five mornings a week. In hospital A, there was an increase in consultation time from 30 min in the beginning to 45 min since the original consultation time had not been sufficient. Sometimes even that length was still not enough for some ML-AC sessions. In hospital B, appointments are set for 30 min; if a pregnant woman needs more time, it is possible for her to make a second appointment. Another issue for successful institutionalization was finding and retaining a **meeting room for the ML-AC consultation**. Both hospitals faced this difficulty. Searching for a room was described by managers and midwives as very challenging, involving multiple discussions with several other units.

**Organizational infrastructure** includes meetings, interprofessional collaboration, and ongoing training on the ML-AC in both hospitals. According to the managers of hospital B, once per year there is a meeting of physicians and midwives to discuss the ML-AC, among other topics. Additionally, 2–3 times / year the ML-AC is on the agenda at the monthly midwives’ meetings to evaluate the implementation and to discuss new topics which may influence the ML-AC. In both hospitals, the midwives can check back with a physician within a short time: “Well, it means that in some cases we think, well she [the pregnant woman] should have seen a physician. Maybe her gynecologist overlooked something, or the woman did not mention this symptom [to the physician]. We are able to present the case to one of our physicians on short notice” (Hospital B-manager 5).

According to managers, midwives, and physicians, interprofessional collaboration is well-established in both hospitals. The existing good collaboration between midwives and physicians supported the implementation and eased maintaining the ML-AC.

Managers and midwives of both hospitals underlined **training and informing all new employees** (physicians and midwives) **on ML-AC** to promote sustainment.

#### Routinization

Routinization pertains to cycles of repeated action in practice and organizational routines [6]. The **structural organization** to routinize ML-AC shows commonalities and differences in the hospitals. One structure was furnished in both hospitals, namely, all appointments were arranged by outpatient services. Differences are especially apparent in the consultation procedure of each hospital. In hospital A, the managers and midwives reported that pregnant women fill out their medical history and the birth plan themselves. During the ML-AC appointment at hospital A, the midwife and the pregnant woman discuss and complete or adapt the medical history and birth plan. In hospital B, the midwife and pregnant woman fill out the medical history and the birth plan together during the ML-AC consultation. In both hospitals, the consultation is noted in the maternal care record, which stays with the pregnant woman. The midwives in both hospitals highlight important issues for other midwives.

The midwives reported in both hospitals that a copy of the medical history and of the birth plan are stored in the delivery unit. Upon arrival, the woman giving birth and the midwife in the delivery unit discuss the most important details of the documents to ensure a mutual understanding. One midwife emphasized: “And then I know already something about her and I can say: “Yes, you were here“, and I can tie in with her and say ‘you told my colleague’ this and that, and we can talk about it. Or if the documentation shows she is scared of something or she had previous experiences, then I talk to her about it, and I ensure that I have a correct understanding. How I perceived it and how she would like me to deal with it. (...) Or to agree that she can be sure that in this situation I will respect this.” (Hospital B-midwife 7).

Differences and similarities in institutionalization and routinization reported by each participant group and level classification (personal, unit or organizational) can be seen in Table 5.

**Table 5:** Institutionalization and routinization of ML-AC as reported by study participants

	Managers	Midwives	Physicians
**Institutionalization** (implies the concretization of organizational infrastructure and integration of a change in subsystems [6])			
**Staff budget** (Unit level)	Hospital A: Overtime, Hospital B: Donations - After about two years increase in number of midwives positions in both hospitals	Separate timetable for midwives offering ML-AC	
**Time slots for ML-AC appointments** (Personal level)	- Hospital A: 30 min. Then 45 min, every day - Hospital B: 30 min. Two days/week - Last trimester of pregnancy	- Hospital A: 30 min. Then 45 min, every week day - Hospital B: 30 min. Two days/week - Last trimester of pregnancy	
**Room for consultation** (Organizational level)	Difficult to obtain a room, lots of discussions	Hospital A: Room without a window	
**Organizational infrastructure** (Organizational level)	- Good collaboration - between midwives and physicians. Consultation with physician on short notice, if needed. - Hospital B: 1/year meeting with physicians, 2–3/year monthly meeting of the midwives	Good collaboration between midwives and physicians. Consultation with physician on short notice, if needed	Good collaboration between midwives and physicians
**Routinization** (pertains to cycles of repeated action in practice and organizational routines [6])			
**Structural organization** (Organizational level)	Appointments by outpatient services Checking appointments Hospital A: Women fill in medical record and birth plan. Midwives add information if necessary Hospital B: Midwives fill in documents together with women during appointment. If necessary, consultation with physicians of outpatient services	If necessary, consultation with physicians of outpatient services. Documentation of consultation in maternal care record. Copy of documents stays in the delivery unit. Content of documents are discussed upon arrival between midwives in the delivery unit and birthing woman	Consultation with physician on short notice, if needed

### Characteristic of sustainability: (3) development

Development is defined as the continual enhancement of users’ abilities and resources to maintain an innovation [29, 32, 33]. As the most important aspect to maintain the ML-AC managers, midwives, and physicians in both hospitals stressed that **reimbursement from health insurance funds** is needed. According to managers, midwives, and physicians, the reimbursement of ML-AC by health insurances would induce hospitals to offer the ML-AC and support them in doing this.

All study participants pointed out that fluctuation of the head physician or head midwife and their willingness to support the ML-AC in the future will have a strong impact on its sustainability. This comment indicates a need to embed the ML-AC into the hospitals’ operational strategies.

On the one hand, offering the ML-AC every day leads to substantial experience of the midwife. On the other hand, routine may occur in a negative sense, with advice being given to all pregnant women in the same way, instead of the midwife tuning in to the special needs of the individual pregnant woman. This danger led to some managers in both hospitals reporting that they offer on-going special training in communication and consultation skills for midwives in the ML-AC team. Also, physicians and managers of both hospitals confirmed the need for the midwives to be committed if the ML-AC is to be offered in the future.

In both hospitals, managers saw the necessity to **extend and adapt to new topics** that arise in the ML-AC. In hospital B, midwives confirmed this need for adaptation to new topics. One midwife emphasized: “And we have to check that we are not stuck with old stuff, but that we are open to new stuff -- what women might also hear out there in prenatal care or do -- and then we have to be able to consult accordingly.” (Hospital B-midwife 2).

In contrast to midwives of hospital B, midwives of hospital A were content with the way things were, pointing out that they have everything they need: “Well, I think if we go ahead just like we do now, everything will be fine.” (Hospital A-midwife 5). Another midwife of hospital A confirmed this view: “I only need the pregnant women and they do come. Well, we have now almost optimal conditions.” (Hospital A-midwife 4).

The results of the managers, ‘midwives’, and physicians´ perspectives show concordance in the need for funding but differences between hospital A and hospital B in the perceived need for extending or adapting the topics of consultation (Table 6).

**Table 6:** Further development of ML-AC as reported by managers, midwives, physicians

	Managers	Midwives	Physicians
**Reimbursement**	Long-term reimbursement through health care system	Permanent midwife positions to offer ML-AC	Financially feasible
**Leadership support**	All managers have to support consultation	Head midwife has to support consultation	Hospital A: Leadership changes can threaten consultation
**Continuing education for ML-AC midwives**	Necessary to support quality of consultations over time	Interest in taking classes	Hospital A: No need for education of midwives
**Development of consultation topics**	Consultation topics have to be adapted over time	Hospital A: No changes necessary Hospital B: Evaluation of new topics on a regular basis	

## Discussion

This study advances the understanding and description of sustainability of the ML-AC as related to (1) benefits, (2) routinization and institutionalization, and (3) development in two hospitals in Germany.

Both hospitals still offer the ML-AC, thus showing that the ML-AC is being maintained, new ways of providing care have become routine, surrounding systems have been transformed in support, and the ML-AC may be developed further [42]. All participants described manifold benefits of the ML-AC on a personal and unit level. The perception of **benefits** by all stakeholders seems to be key for maintaining sustainability. The described benefits were achieved through the initial implementation [43] and the following adaptations during routinization and institutionalization. If important stakeholders of an intervention perceive no benefits, maintaining that intervention is problematic. Especially administrative support and resources are crucial for long-term sustainability [14, 44, 45]. For managers, their support can be ensured if benefits are perceived on an organizational level, such as saving time.

**Routinization and institutionalization** are rarely described [46]. The participants of this study underlined changes and adaptations that were important to sustaining the ML-AC over a period of two to three years. These changes, e.g., allocation of working time, are essential to program survival [29].

Hospitals implementing the ML-AC will need to be aware that adaptations of the NES are necessary to accomplish routinization and institutionalization. Routinization and institutionalization are adaptation processes that are on-going in both hospitals, indicating further effort that is needed to sustain the intervention. Also, the description of institutionalization and routinization shows why training alone is not enough to implement and sustain an intervention such as the ML-AC. Particularly establishing a structural organization means establishing processes and cooperation with other units, departments, and occupational groups. The effort and time needed for institutionalization and routinization are rarely planned and funded, with a subtle expectation that the work can be done on top of the tight workloads of managers, midwives, and physicians. This is a possible explanation of and can contribute to implementation and sustainability failure.

Also, further **development** might be critical for the sustainability of an intervention. Awareness needs to be raised that evaluating and possibly adapting an intervention are essential and work needs to continue once an organization has succeeded in implementing the ML-AC. To evaluate an intervention such as the ML-AC, there has also to be clearly defined core and adaptable criteria of the intervention [1].

Currently, German hospitals offering ML-AC at an NES level must finance the ML-AC with the budget they have. There is no reimbursement through the health care system, thus possibly impeding long-term sustainability. The importance of health care financing seems to be essential for sustainability in general [14, 45, 47, 48]. This study contributes to the understanding of the role of initial funding and funding over two to three years, e.g., a midwife full time equivalent position, which is rarely described in published sustainability research [46]. We found that, although both hospitals initially financed the ML-AC from various sources, permanent reimbursement by health insurance funds is the only way to secure long-term sustainability. To achieve permanent funding, further research is necessary to examine efficacy and effectiveness and to undertake health economic evaluations of ML-AC. Apart from funding stability Hunter et al. (2015) found also political support, e.g., rules and regulations that organizations require, important for sustainability [48].

According to Leffers & Mitchel (2011), one key predictor for sustainability is on-going assessment across all organizational levels (person, unit, and organization) of the hospital [43]. Another key predictor is the collaboration among stakeholders of the intervention [43] which was seen between management, midwives, and physicians in our study. Other key predictors are appropriate resources, and on-going evaluation [43, 45, 49]. On-going evaluation seems to be crucial, in addition to staff budget, and implies that sustainability is not just given but is a process that has no defined end.

### Limitations

There are several limitations of this study that have to be considered. Only study participants from two hospitals that had implemented ML-AC were interviewed. It is possible that only staff who had a positive attitude to ML-AC chose to participate in the study. Pregnant women who had ML-AC potentially gave socially desirable answers since these women will come back to the hospital to give birth. Perhaps theoretical saturation of the data is not established due to the limited number of interview partners. Even though participants were asked specifically about ML-AC, some extended their explanations to all elements of the NES to promote physiological childbirth.

## Conclusion

In summary, it is essential to monitor and evaluate benefits, routinization and institutionalization, and further development if an intervention is to be sustained. Table 7 offers some recommendations to promote the sustainability of the ML-AC.

**Table 7:** Recommendations to achieve sustainability of ML-AC

**Recommendations to achieve sustainability of ML-AC**	
*√* Evaluate perceived benefits of all stakeholders, including the pregnant women. *√* Establish and keep institutionalization of ML-AC through ○ securing of staff budget, ○ timeslots for appointments, ○ meetings for evaluation on a regular basis, ○ a designated room and ○ well established interprofessional collaboration. *√* Establish and keep routinization through ○ agreed upon structural organization of e.g., appointments by outpatient services, ○ information flow and ○ documentation *√* Embed ML-AC into the operational guidelines of the hospital. *√* Secure long-term reimbursement. *√* Secure support of management, midwives and physicians for ML-AC. *√* Offer continuing education for midwives offering ML-AC. *√* Evaluate and adapt topics of ML-AC on a regular basis with all midwives in the hospital.	

This study uses conceptual and operational definitions for sustainability and adds to the understanding of the relationship between the service system context and sustainability [9]. It describes health intervention sustainability and addresses thus requisitions associated with sustainability research [9]. Our findings suggest an intervention has neither a defined end of implementation nor a distinct beginning of sustainability. The process is more a transition between the two as described by Fleiszer et al. (2015) [6]. Planning, monitoring, and evaluating all three characteristics of sustainability are probably necessary for long-term sustainability.

## Data Availability

The datasets generated and/or analyzed during the current study are not publicly available due to German data protection regulation and requirements of ethics approval but are available from the corresponding author on reasonable request.
